# Non-canonical d-xylose and l-arabinose metabolism via d-arabitol in the oleaginous yeast *Rhodosporidium toruloides*

**DOI:** 10.1186/s12934-023-02126-x

**Published:** 2023-08-03

**Authors:** Paul A. Adamczyk, Samuel T. Coradetti, John M. Gladden

**Affiliations:** 1 Agile Biofoundry, Emeryville, CA USA; 2 Sandia National Laboratories, Livermore, CA USA; 3 Present Address: United States Department of Agriculture, Agricultural Research Service, Ithaca, NY USA; 4 Joint BioEnergy Institute, Emeryville, CA USA; 5 Sandia National Laboratories, DOE Agile Biofoundry, 5885 Hollis Street, Fourth Floor, Emeryville, CA 94608 USA

**Keywords:** *Rhodosporidium toruloides*, *Rhodotorula*, d-xylose metabolism, l-arabinose, d-arabitol, Xylulokinase, l-ribulose, Xylitol, l-arabitol, Pentose metabolism

## Abstract

*R. toruloides* is an oleaginous yeast, with diverse metabolic capacities and high tolerance for inhibitory compounds abundant in plant biomass hydrolysates. While *R. toruloides* grows on several pentose sugars and alcohols, further engineering of the native pathway is required for efficient conversion of biomass-derived sugars to higher value bioproducts. A previous high-throughput study inferred that *R. toruloides* possesses a non-canonical l-arabinose and d-xylose metabolism proceeding through d-arabitol and d-ribulose. In this study, we present a combination of genetic and metabolite data that refine and extend that model. Chiral separations definitively illustrate that d-arabitol is the enantiomer that accumulates under pentose metabolism. Deletion of putative d-arabitol-2-dehydrogenase (RTO4_9990) results in > 75% conversion of d-xylose to d-arabitol, and is growth-complemented on pentoses by heterologous xylulose kinase expression. Deletion of putative d-ribulose kinase (RTO4_14368) arrests all growth on any pentose tested. Analysis of several pentose dehydrogenase mutants elucidates a complex pathway with multiple enzymes mediating multiple different reactions in differing combinations, from which we also inferred a putative l-ribulose utilization pathway. Our results suggest that we have identified enzymes responsible for the majority of pathway flux, with additional unknown enzymes providing accessory activity at multiple steps. Further biochemical characterization of the enzymes described here will enable a more complete and quantitative understanding of *R. toruloides* pentose metabolism. These findings add to a growing understanding of the diversity and complexity of microbial pentose metabolism.

**Supplementary Information:**

The online version contains supplementary material available at 10.1186/s12934-023-02126-x.

## Introduction

Economically viable biorefineries upgrading lignocellulosic biomass to value-added products using microbial platforms will need to efficiently utilize all four major sugar monomers—d-glucose, d-xylose, d-mannose, and l-arabinose [1]. While d-glucose is the preferred substrate for most organisms, many have little to no capacity to metabolize d-xylose, the second largest component of biomass [2–4]. A deeper understanding of pentose catabolism in diverse organisms is required in order to engineer strains with utilization efficiency on par with that of d-glucose.

**Fig. 1:**
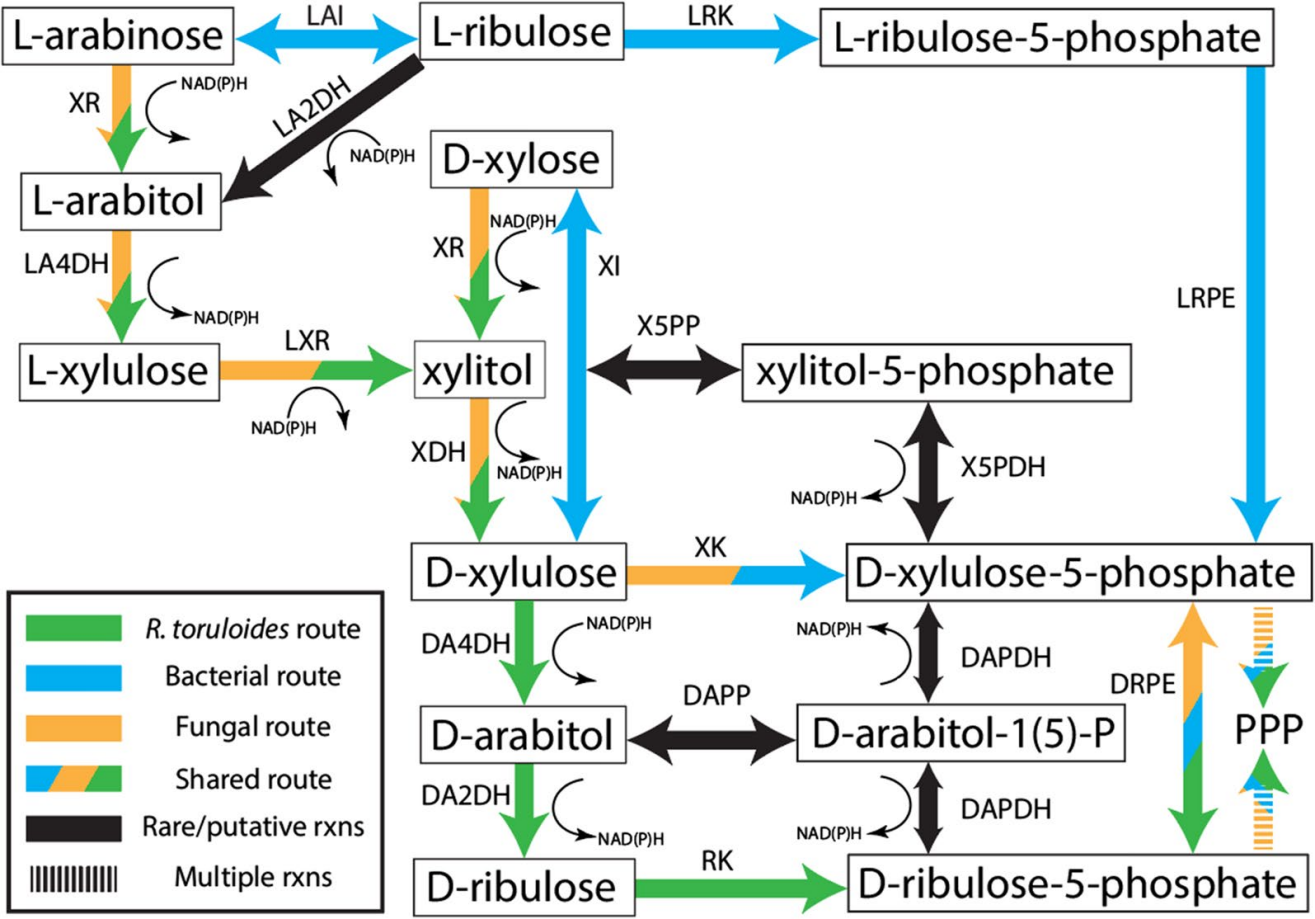
Canonical fungal and bacterial l-arabinose and d-xylose metabolism are additionally overlaid. Included are most known or putative routes for d-arabitol and xylitol interconversion. Cofactor annotations were left generally as NAD(P)H instead of species-specific. Please refer to Fig. 14 for gene-protein-reaction rules inferred from this study. *XR* broad-substrate-specificity d-xylose reductase, *LA4DH* l-arabitol-4-dehydrogenase, *LA2DH* l-arabitol-2-dehydrogenase, *LXR* l-xylulose reductase, *LAI* l-arabinose isomerase, *LRK* l-ribulose kinase, *LRPE* l-ribulose-5-phosphate 4-epimerase, *XI* d-xylose isomerase, *XDH* xylitol dehydrogenase, *DA4DH* d-arabitol-4-dehydrogenase, *DA2DH* d-arabitol-2-dehydrogenase, *RK* d-ribulose kinase, *XK* d-xylulose kinase, *DAK* d-arabitol kinase, *X5PP* xylitol-5-phosphate phosphatase, *Ru5P* d-ribulose-5-phosphate, *Xu5P* d-xylulose-5-phosphate, *DAPDH* d-arabitol-phosphate dehydrogenase, *DRPE* d-ribulose-5-phosphate 3-epimerase, *DAPP* d-arabitol phosphate phosphatase, *X5PDH* xylitol-5-phosphate dehydrogenase, *PPP* pentose phosphate pathway, d-*arabitol-1(5)-P* indicates either d-arabitol-1-phosphate or d-arabitol-5-phosphate, depending on the precursor it was converted from, *rxns* reactions

*Rhodosporidium toruloides* (*Rhodotorula toruloides*) is an oleaginous yeast capable of consuming many diverse substrates (including waste office paper, cassava starch, and macroalgae hydrolysate) [5–7]. It is able to tolerate and degrade potential inhibitors in biomass hydrolysates and non-traditional carbon streams (including furfural, 5-HMF, vanillin, syringaldehyde, vanillic acid, levulinic acid, and acetic acid) [8, 9], making it a prime candidate for Acetyl-CoA (AcCoA)-derived bioproducts such as lipids and terpenes. However, *R. toruloides* suffers from appreciably slower d-xylose growth relative to d-glucose [10] and an understudied pentose metabolism.

 d-xylose and l-arabinose metabolism occurs primarily via cofactor-dependent oxidoreductase and redox-neutral isomerase pathways in eukaryotes and prokaryotes, respectively [11]. The d-xylose oxidoreductase pathway includes reduction via d-xylose reductase (XR), xylitol oxidation via xylitol dehydrogenase (XDH), and phosphorylation to d-xylulose-5-phosphate (Xu5P) via d-xylulose kinase (XK). Similarly, eukaryotic l-arabinose metabolism begins with l-arabinose reduction via l-arabinose reductase (or XR), l-arabitol oxidation via l-arabitol-4-dehydrogenase (LA4DH), and l-xylulose reduction via l-xylulose reductase (LXR). Figure 1 illustrates the 4 pathways describing catabolism of the major pentose monomers of deconstructed biomass. However, deviations from the norm exist (e.g., the anaerobic fungus *Piromyces* sp. strain E2 uses d-xylose isomerase (XI) and XK to metabolize d-xylose [12]).

A recent study of multi-omics analysis and metabolic model curation surmised—with RNAseq, proteomics, and functional genomics—that *R. toruloides* possesses non-canonical pentose metabolism through d-arabitol, d-ribulose, and d-ribulose-5-phosphate (Ru5P) [13] (Fig. 1). However, R. toruloides has a putative XK (encoded by RTO4_16850), but RNA transcript levels are near the limit of detection and no XK peptides were detected on both pentose and d-glucose [13]. Simultaneously, putative genes RTO4_9990 and RTO4_14368 (encoding d-arabitol-2-dehydrogenase (DA2DH) and d-ribulose kinase (RK), respectively) were strongly expressed at the peptide level and mutants in these genes had strong growth defects on nearly all pentose substrates examined, whereas mutants in XK had no growth defects in any condition tested [13]. Supporting these data, Jagtap et al. reported that *R. toruloides* excretes d-arabitol with d-xylose as the sole carbon source; however, they postulated that d-arabitol is a dead-end overflow metabolite to maintain redox homeostasis under high d-xylose utilization conditions alongside traditional flux through XK [14]. Under this model, deletion of RTO4_9990 (putative DA2DH) should have a minor effect on d-xylose growth, with eventual complete or near-complete growth recovery, contradicted by Kim et al. [13]. An additional study by Jagtap et al. with metabolomics and transcriptomics on several carbon sources recapitulates and reinforces the findings of Kim et al., showing no XK transcription on d-xylose with a simultaneous upregulation of the putative alternative route via d-arabitol [13, 15]. The authors updated the model in congruence with the findings of Kim et al.; however, no further pathway characterization or verification was performed.

Production of d-arabitol (and other polyols) from d-glucose or glycerol (with Xu5P or Ru5P precursors) is evident in many fungi [16–21], or l-arabitol from l-arabinose [22, 23], hinting that other fungi might possess alterations to canonical pentose metabolism. Production of d-arabitol from d-xylose or l-arabinose is less characterized, although some studies exist.

For example, Saha et al. showed that *Zygosaccharomyces rouxii* is able to produce d-arabitol and/or xylitol from many carbon sources including d-xylose, d-glucose, d-xylulose, d-fructose, and d-mannose, proposing an unverified pentose model accounting for their observations [24, 25]. A previous study of purified extracts of a different *Z. rouxii* strain fed [5-14C] d-xylulose demonstrated NADH-linked DA4DH reduction of d-xylulose to d-arabitol [26], with no activity on d-arabitol, but some activity on xylitol (XDH activity), suggesting putative DA4DH is irreversible—either intrinsically so or due to thermodynamic constraints of the tested condition. From other work, it was concluded that DA2DH activity is responsible for production of d-arabitol from d-glucose [27]. Unfortunately, d-arabitol production from d-xylose was not assessed, preventing a definitive understanding in *Z. rouxii*, although production seems likely via simultaneous DA4DH and DA2DH activity. In the fungus *Pichia anomala* d-arabitol, xylitol, and ribitol is produced solely from d-xylose, in addition to d-arabitol and xylitol solely from d-glucose [28]. Zhang et al. characterized the putative arabitol dehydrogenase in vitro showing reversible DA4DH and irreversible XDH (d-xylulose-forming only) activities, both NADH-dependent, but with highest activity on d-arabitol. However, there was no verification of their theoretical model of *P. anomala* pentose metabolism, so details of d-xylose metabolism remain unclear.  A study of *Candida maltosa* grown on d-xylose mother liquor produced mostly xylitol, with a small fraction of d-arabitol [29, 30]. The authors then tested for growth solely on d-arabitol without observing xylitol production. They surmised an irreversible reaction of d-xylulose reduction (to d-arabitol) and proposed a potential d-xylose catabolic pathway accounting for d-arabitol production that includes both canonical (XR, XDH, XK) and non-canonical routes (XR, XDH, DA4DH, DA2DH). However, no in-depth characterization of the pathway or responsible genes was carried out to confirm this model. *Candida arabinofermentans* and *Pichia guilliermondii* fed 13C-labeled l-arabinose produced both labeled d-arabitol and labeled l-arabitol, as detected via NMR. However, the authors inferred that that d-arabitol was produced from d-ribulose via the PPP as opposed to directly from d-xylulose, and hypothesized that this only serves as a means to regenerate NAD^+^ during oxygen-limiting conditions rather than a mainstay for pentose metabolism [31, 32]. Additional examples and a more comprehensive survey of arabitol and other polyols produced by various yeasts can be found elsewhere [20, 33–39].

Taken together, data from different fungi suggest a diversity of polyol metabolism linked to pentose catabolism, though in most cases the data are fragmented with uncertainty as to which polyols are intermediates in the main pathway, and which may be side products. Also, while previous work by Kim et al. [13] provides solid evidence that a non-canonical pathway exists in *R. toruloides* and identifies several enzymes involved, high-throughput fitness studies are limited in their precision, especially for cases of overlapping enzyme function. In order to engineer *R. toruloides* for optimal pentose conversion, a more complete picture of the catabolic pathway and the enzymes mediating each step is required. To this end, we systematically probed the functions of each of the major putative *R. toruloides* pentose genes via genomic deletions and selective complementation by heterologous XI and XK. Growth phenotyping on representative pentose substrates, metabolite profiling, and enantiomer determination of arabitol accumulated in the growth medium were employed to piece together a clear picture of this unusual pentose metabolism and further validate previous high-throughput observations. Lastly, we show that the pentose pathway is functionally redundant at nearly every step and explore an unusual substrate-specific bypass to our proposed pentose metabolic model.

## Results

### Growth phenotyping of putative pentose catabolic enzyme deletions

**Fig. 2:**
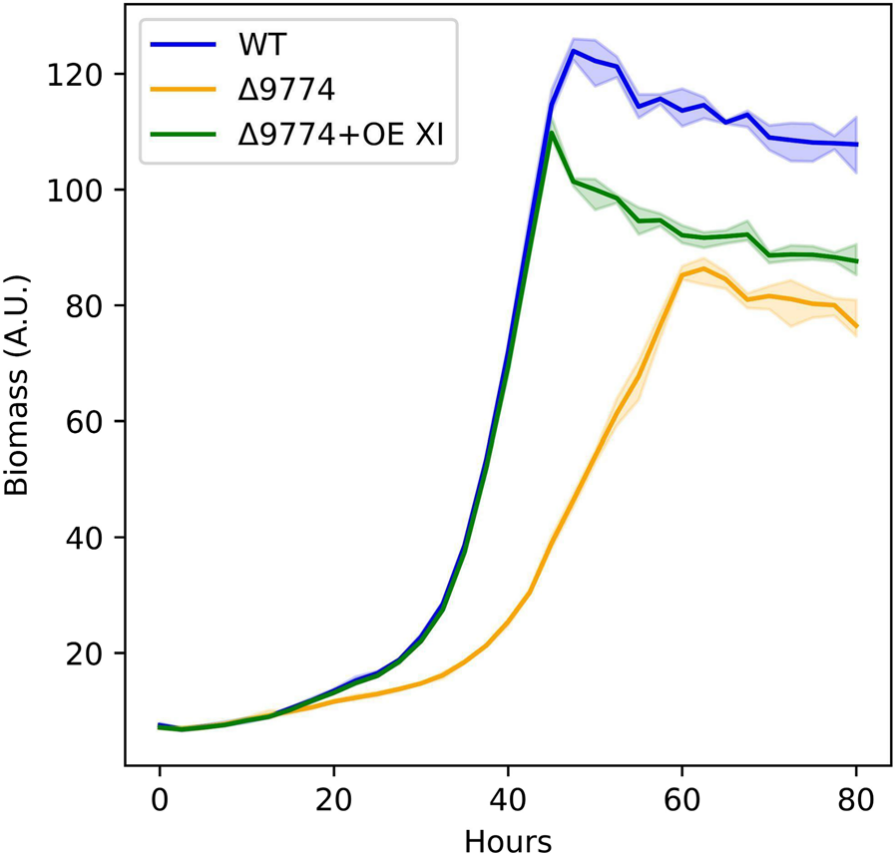
Growth of WT vs putative d-xylose reductase deletion complemented with XI on 40 g/L d-xylose medium. Solid lines are the average of 3 biological replicates; shaded regions indicate 100% percentile intervals; OE: overexpression; A.U.: Arbitrary Units

As in most eukaryotes, the first step in d-xylose metabolism in *R. toruloides* is likely via an XR as opposed to an XI [40]. Protzko et al. found a putative l-glyceraldehyde and general pentose reductase (encoded by RTO4_9774) critical for d-galacturonic acid metabolism. In vitro enzyme activity assays showed that substrates of the reductase include l-glyceraldehyde, l-arabinose, and d-xylose, and NADPH. This broad substrate specificity is not unusual amongst fungi [41, 42]. Although the preferred substrate of the reductase is not d-xylose, we still assessed its role in d-xylose metabolism. An RTO4_9774 deletion resulted in diminished, but not abolished, growth on d-xylose as the sole carbon source, indicating XR redundancy (Fig. 2). Alignment of characterized fungal XRs from *Aspergillus niger* and *Trichoderma reesei* to *R. toruloides* suggests many additional candidates for this activity.

There is no evidence that *R. toruloides* has a functional XI, allowing us to employ XI in our investigation of the pentose assimilation pathway. To test its functionality, codon-optimized XI (from *Lachnoclostridium phytofermentans*) was randomly integrated (via *Agrobacterium tumefaciens*-mediated transformation (see methods)) into ΔRTO4_9774 (i.e., ΔRTO4_9774 + OE XI), driven by a strong promoter (*Rhodotorula graminis* Tef1; Fig. 2), recovering growth of the deletion mutant. This indicates that d-xylulose (the product of XI) is likely a native metabolite of pentose metabolism, and that the rate-limiting step of pentose metabolism is probably either downstream of d-xylulose (provided sufficient *L. phytofermentans* XI expression and activity) or at the point of transport into the cell.

**Fig. 3:**
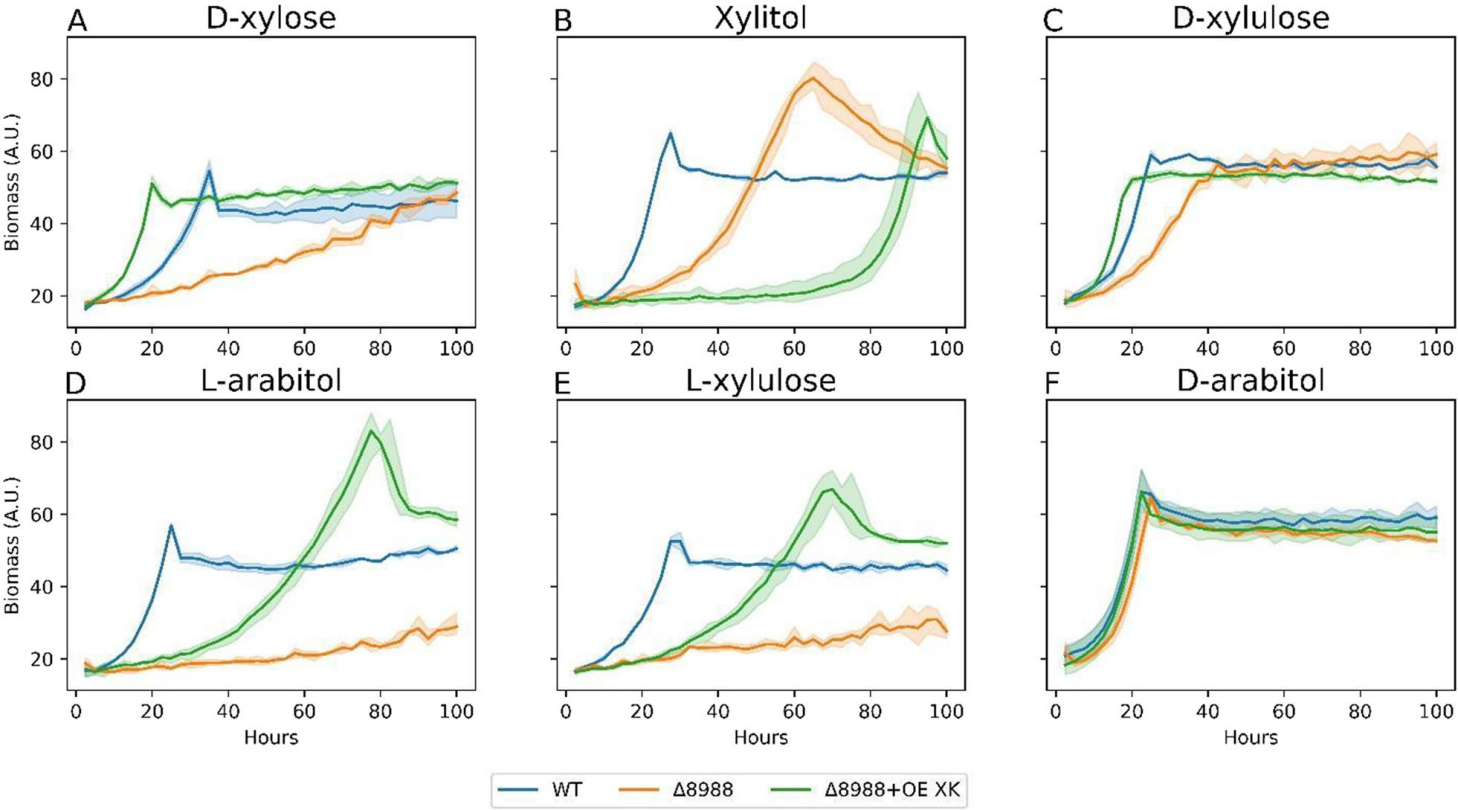
Growth curves of WT, ΔRTO4_8988, ΔRTO4_8988 + OE XK on 5 g/L per sugar. Solid lines are the average of 3 biological replicates; shaded regions indicate 100% percentile intervals

Following reduction of d-xylose, xylitol is likely oxidized to d-xylulose via XDH (Fig. 1). RTO4_8988 mutants have considerable fitness defects on multiple carbon sources (l-arabinose, l-arabitol, l-lyxose, xylitol), and modest defects on d-xylose, d-xylulose [13]. Thus, RTO4_8988 likely plays a promiscuous role in l-arabitol and d-xylose metabolism. Orthologous sequences from the filamentous fungi, *T. reesei*, with empirical data obtained from cell-free extracts and purified enzyme assays include an NADPH-dependent d-mannitol 2-dehydrogenase encoded by *lxr1* [43], and an NADPH-dependent LXR encoded by *lxr3* [44], the latter showing promiscuous polyol-forming activity on many substrates in addition to l-xylulose—notably d-xylulose [44]. Additionally, characterized LXR1 from *A. niger* shows weak similarity to only a single gene, RTO4_8988 [45, 46]. Unsurprisingly, ΔRTO4_8988 grown on select pentose substrates shows multiple growth defects in our proposed pentose metabolism model (Fig. 3). Namely, growth defects are observed on d-xylose, xylitol, d-xylulose, l-xylulose, or l-arabitol. The progressively worsening growth defects of RTO4_8988 mutants as the carbon source is moved upstream in the pentose utilization pathway definitively supports RTO4_8988 d-arabitol-4-dehydrogenase (DA4DH) activity and possibly XDH, LXR activities.

Under canonical fungal metabolism, d-xylulose is converted to Xu5P via XK (Fig. 1). Comparative sequence analysis to characterized fungal XKs reveals that *R. toruloides* RTO4_16850 likely encodes an XK [47, 48]; however, under no conditions tested were RTO4_16850 peptides detected or any fitness defects observed upon deletion via RB-TDNAseq fitness profiling [13]. Either regulatory mechanisms, improper conditions, coding mutations, or cofactor balancing are suppressing expression and activity. To explore the functionality of RTO4_16850, a plasmid driven by a strong native promoter (P14 Tef1; [49]) expressing native RTO4_16850 sequence was randomly integrated into ΔRTO4_9990 (deletion without growth on d-xylose), and screened for growth on d-xylose as the sole carbon source. No growth was observed in any of the 48 transformants.

**Fig. 4:**
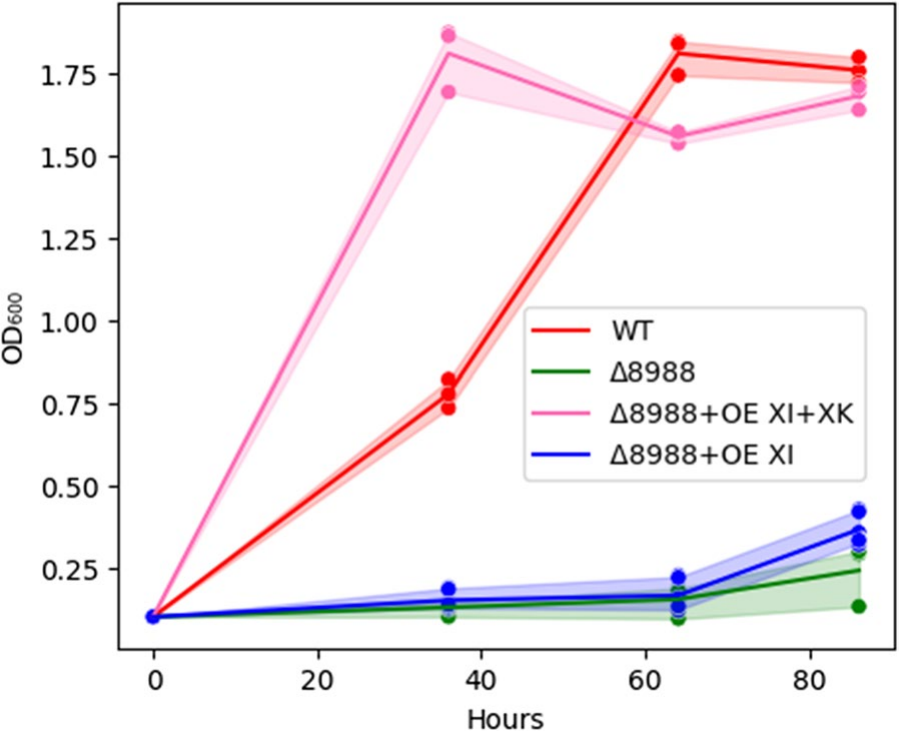
WT, ΔRTO4_8988, ΔRTO4_8988 + OE XI + XK, ΔRTO4_8988 + OE XI biomass growth curves on 5 g/L d-xylose. Solid lines are the average of 3 biological replicates; shaded regions indicate 100% percentile intervals

With strong evidence of no native XK functionality, XK from *A. niger* was codon optimized, overexpressed on a plasmid driven by the strong *Rhodotorula graminis* Tef1 promoter, and randomly integrated in ΔRTO4_8988 (i.e., ΔRTO4_8988 + OE XK). Growth on four of five sugars with observed growth deficits in ΔRTO4_8988 was partially or fully recovered upon XK complementation (Fig. 3). Most notable is the vastly improved growth of ΔRTO4_8988 + OE XK relative to WT on d-xylose (Fig. 3A), implying pentose metabolism is possibly rate limited downstream XDH, and RTO4_8988 may encode minor XDH activity. Interestingly, ΔRTO4_8988 + OE XK has an exacerbated growth deficit on xylitol relative to ΔRTO4_8988, potentially due to unbalanced redox homeostasis in this condition. ΔRTO4_8988 + OE XK grown on l-arabitol and l-xylulose shows partial recovery relative to WT, supporting RTO4_8988 LXR activity (Fig. 3D–E). As expected, the d-xylulose growth deficit from loss of RTO4_8988 DA4DH activity is complemented by ΔRTO4_8988 + OE XK (Fig. 3C). Growth of ΔRTO4_8988 + OE XK on d-arabitol is not impacted by XK expression, possibly due to reaction irreversibility of DA4DH, thermodynamic constraints, or cofactor/redox imbalance. We further explored the effects of XI and XK overexpression in ΔRTO4_8988 on d-xylose via random integration with the *Rhodotorula graminis* Tef1 promoter (Fig. 4). ΔRTO4_8988 + OE XI has an identical growth rate to ΔRTO4_8988; however, once XK is overexpressed with ΔRTO4_8988 + OE XI (Fig. 4), growth surpasses WT (similar to Fig. 3A), indicating XR and XDH are not rate limiting. The lack of XI improving growth is similar to Fig. 2 (ΔRTO4_9774 + OE XI).

**Fig. 5:**
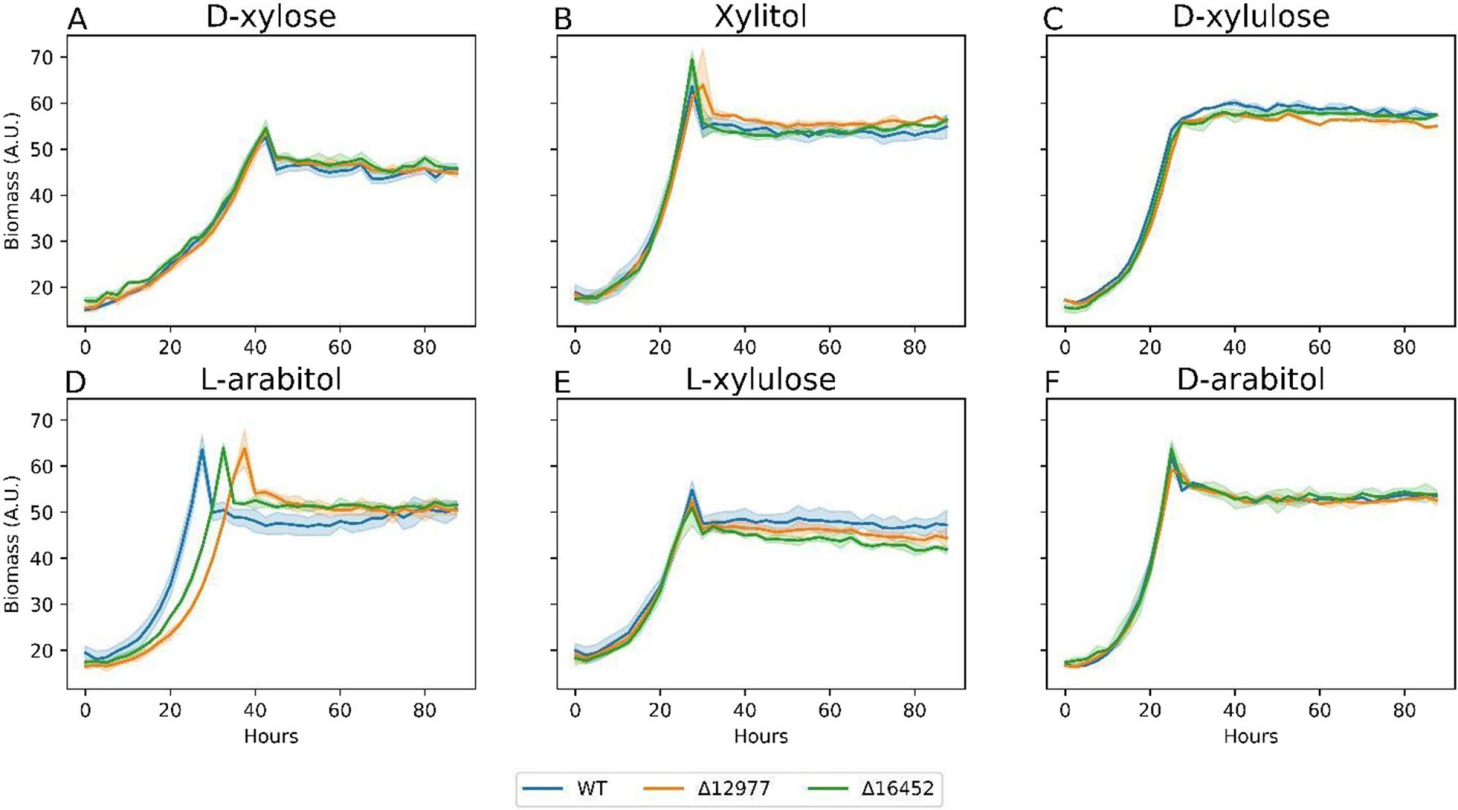
WT, ΔRTO4_12977, ΔRTO4_16452 biomass growth curves on 5 g/L per sugar. Solid lines are the average of 3 biological replicates; shaded regions indicate 100% percentile intervals

Cross referencing RB-TDNAseq mutant fitness, another putative XDH—RTO4_16452—has moderate fitness defects upon deletion on xylitol and l-arabitol, with elevated expression on d-xylose and l-arabinose [13]. Characterized orthologs from *Aspergillus oryzae*, *Candida tropicalis*, *Arxula adeninivorans*, and *A. niger* exhibit NAD^+^-dependent XDH activity [46, 50–53]. However, when ∆RTO4_16452 was grown on 6 pentose intermediates, a growth defect was only observed on l-arabitol, supporting LA4DH activity (Fig. 5). Various fungi have LA4DHs that function as promiscuous XDHs. For example, in *A. oryzae*, LA4DH is active on several polyols—ribitol, l-arabitol, xylitol—indicating redundant XDH activity, with *la4dh* or *xdh* deletions equally improving xylitol titers [54, 55]. Seiboth et al. showed *T. reesei* XDH NAD^+^-dependent activity on xylitol and d-xylulose, but inactivity on l-arabitol and l-arabinose, with its deletion complemented by LA4DH [56]. Notably, RTO4_16452 protein alignment to putative XDH RTO4_8988 shows no similarity, but significant similarity to RTO4_12977 (i.e., 70% coverage and 41% identity)—another putative LA4DH and potential XDH.

RTO4_12977 shows significant expression on d-xylose and l-arabinose and, when deleted, moderate fitness defects on l-arabitol, l-lyxose, and l-arabinose [13]. Growth of ΔRTO4_12977 on 6 pentose sugars supports possible minor LA4DH function with no obvious growth detriments on d-xylose, xylitol, or l-xylulose (Fig. 5). Orthologous fungal sequences indicate LA4DH with partial XDH activities (NAD^+^-linked) [54, 55, 57–60]. Interestingly, a sequence alignment within the *R. toruloides* genome shows that RTO4_12977 is strikingly similar to RTO4_12974 (99% coverage and 91% identity); however, ΔRTO4_12974 showed no growth deficits on any of the 6 pentose sugars. Furthermore, RTO4_12974 data from Kim et al. shows no fitness defects upon deletion on any pentose intermediate as well as no transcripts on either d-xylose or l-arabinose [13]. Proteomics data displays an abundance of RTO4_12974 peptides, but due to the very high sequence similarity with RTO4_12977, unique peptides are sparse. Therefore, RTO4_12974 is likely a pseudogene.

In the absence of an endogenous functional XK in *R. toruloides*, d-xylulose is likely converted to d-arabitol via a DA4DH—a critical step distinguishing canonical vs non-canonical pentose metabolism (Fig. 1). Only two characterized fungal DA4DHs exist, one from the rust fungus, *Uromyces fabae* [61], but no clear ortholog is evident in the *R. toruloides* genome. Heterologous expression of ARD1 from *U. fabae* in *Saccharomyces cerevisiae* extracts showed NADPH-linked conversion of both d-xylulose and d-ribulose to d-arabitol in isolation (demonstrating DA2DH and DA4DH activities), not in context with global pentose metabolism, so it is unclear how d-xylose metabolism proceeds in *U. fabae*, although the authors posit d-arabitol is derived from PPP intermediates. Weak potential orthologs consist of ~ 55% coverage and ~ 30% identity relative to *U. fabae* DA4DH, including RTO4_12977, RTO4_16452, and a pair of similar, putative alcohol/2,3-butanediol dehydrogenases RTO4_9634 and RTO4_13641. The second characterized NADH-dependent DA4DH with additional activity as an XDH is from *P. anomala* [28]. Alignment to the *R. toruloides* genome shows ~ 60% coverage and ~ 40% identity to RTO4_9990 and RTO4_8988. RTO4_8905 and RTO4_9837 were two additional putative DA4DHs assigned in Kim et al. [13]; however, single deletions had no growth deficits in any condition tested.

**Fig. 6:**
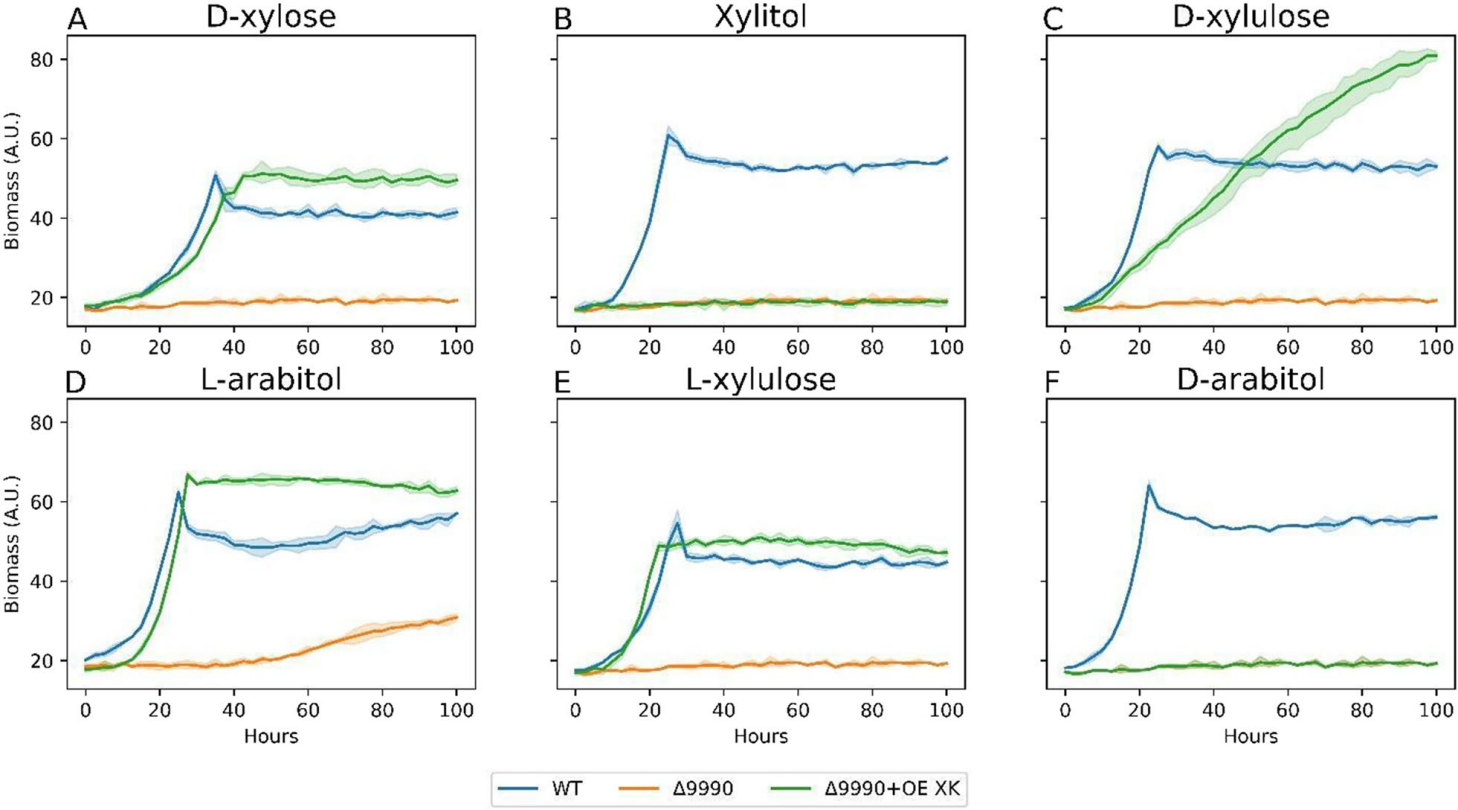
Growth curves of WT, ΔRTO4_9990, ΔRTO4_9990 + OE XK on 5 g/L per sugar. Solid lines are the average of 3 biological replicates; shaded regions indicate 100% percentile intervals

**Fig. 7:**
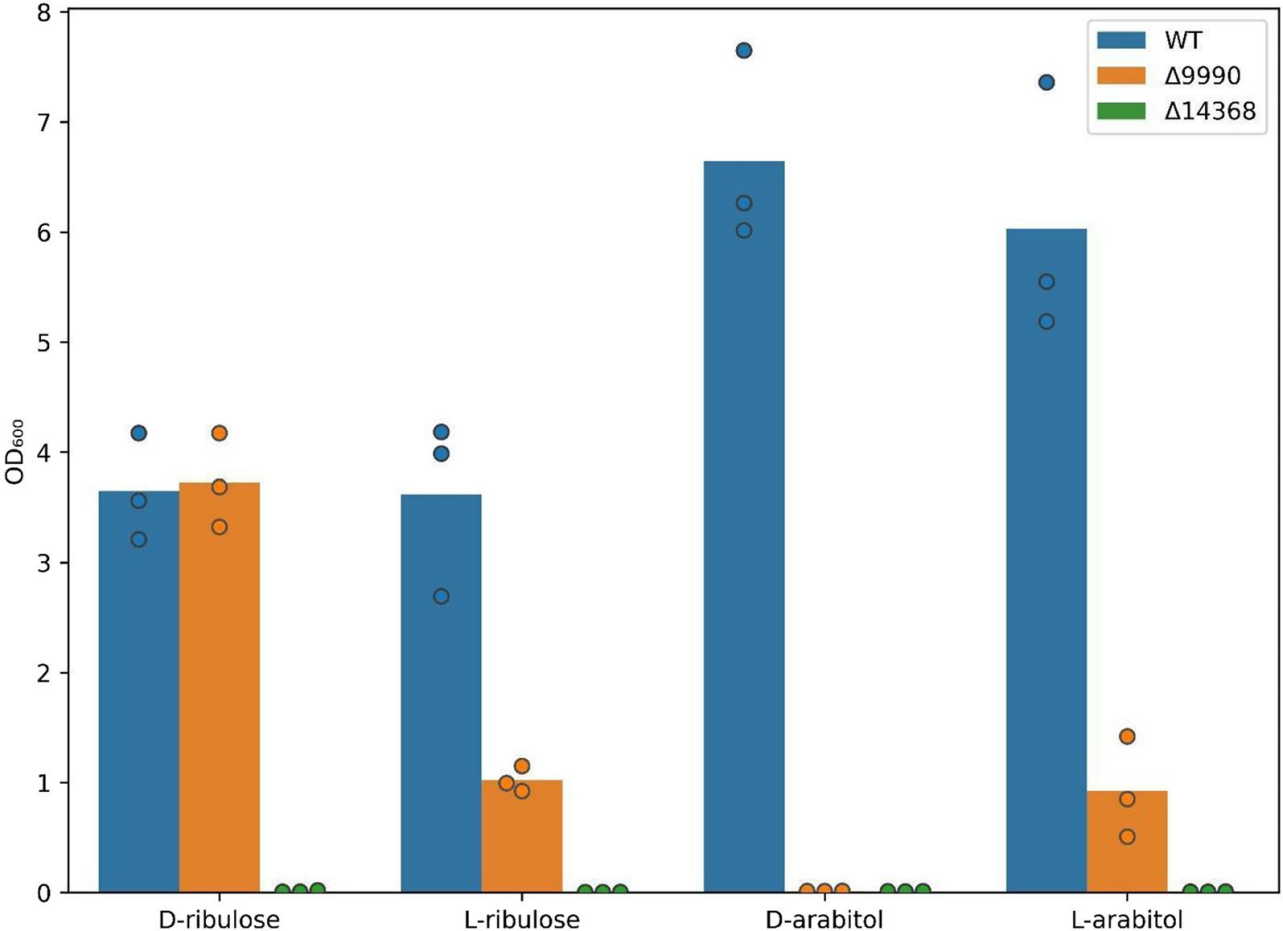
Averaged biological triplicate, 240-h end-point OD_600_ measurements of WT, ΔRTO4_9990, ΔRTO4_14368 grown on 10 g/L per sugar

Following d-xylulose reduction, d-arabitol is probably converted to d-ribulose via DA2DH (Fig. 1). In RB-TDNAseq fitness profiling, ΔRTO4_9990 had no growth defect on d-ribulose, but had severe fitness defects on l-arabitol, d-xylose, l-lyxose, l-arabitol, xylitol, d-xylulose, and d-arabitol [13]. This strongly implies RTO4_9990 encodes a DA2DH. In vitro characterizations of orthologs from *C. tropicalis, Pichia stipitis, P*. *anomala*, *Gluconobacter oxydans, C. albicans*, *Ambrosiozyma monospora* predict mainly an NAD^+^-dependent DA2DH, followed by possible DA4DH, XDH, and LXR activities [20, 28, 62–65]. Interestingly, significant protein alignment (93% coverage and 40% identity) of RTO4_9990 only exists with RTO4_8988 and no other *R. toruloides* genes, highlighting potential functional overlap. We screened ΔRTO4_9990 on 8 pentose substrates and observed mild growth on l-arabitol, l-ribulose, but robust growth on d-ribulose, indicative of DA2DH activity (Figs. 6 and 7). This no-growth phenotype is mirrored in *C. albicans*, whereby deletion of putative DA2DH eliminates growth on d-arabitol and d-arabinose [20]. Next, we overexpressed *A. niger* XK in ΔRTO4_9990 and tested for growth complementation on 6 pentose substrates (Fig. 6). Identical or better growth relative to WT was observed on l-xylulose, l-arabitol, and d-xylose, signifying RTO4_9990 does not have major roles upstream d-xylulose. No growth complementation was observed on xylitol, despite full growth recovery on d-xylose, l-arabitol, and l-xylulose, possibly attributed to cofactor or redox imbalance (similar to ΔRTO4_8988 + OE XK; Fig. 3B). Near complete growth recovery is seen on d-xylulose, demonstrating *R. toruloides* does not have an active native XK under these conditions. No growth complementation was observed on d-arabitol, possibly due to cofactor/redox imbalance, thermodynamic or enzymatic irreversibility—similar to *Z. rouxii* DA4DH [26]—or RTO4_9990 also encodes DA4DH activity.

**Fig. 8:**
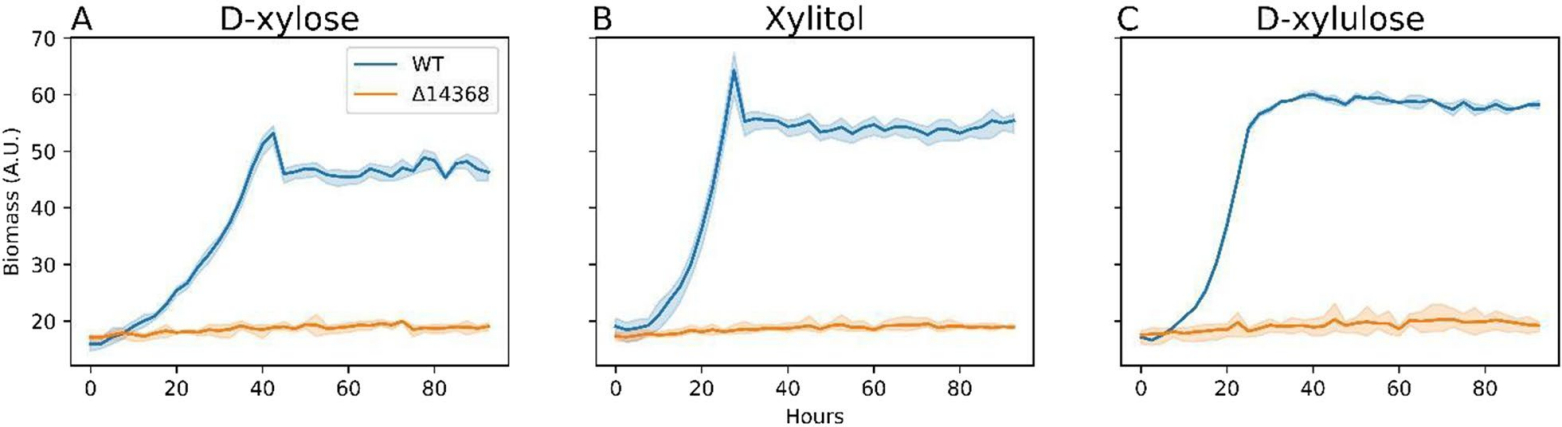
Growth curves of WT, ΔRTO4_14368 on 5 g/L per sugar. Solid lines are the average of 3 biological replicates; shaded regions indicate 100% percentile intervals

After d-arabitol oxidation, d-ribulose is most likely converted to Ru5P (Fig. 1) via putative RK encoded by RTO4_14368, orthologous to characterized *S. cerevisiae* RK [66]. Like RTO4_9990, RTO4_14368 is highly upregulated on pentose sugars l-arabinose and d-xylose, but unlike ΔRTO4_9990, ΔRTO4_14368 exhibits major fitness defects on d-ribulose in addition to other pentoses [13]. We constructed ΔRTO4_14368 and observed growth across 4 pentose substrates (l-ribulose, d-ribulose, l-arabitol, d-arabitol; Fig. 7) in addition to d-xylulose, xylitol, and d-xylose (Fig. 8). ΔRTO4_14368 exhibited no growth on any of the 7 substrates tested, implying that all pentose metabolism proceeds through RK.

### Metabolite excretion profiles of key pentose mutants

**Fig. 9:**
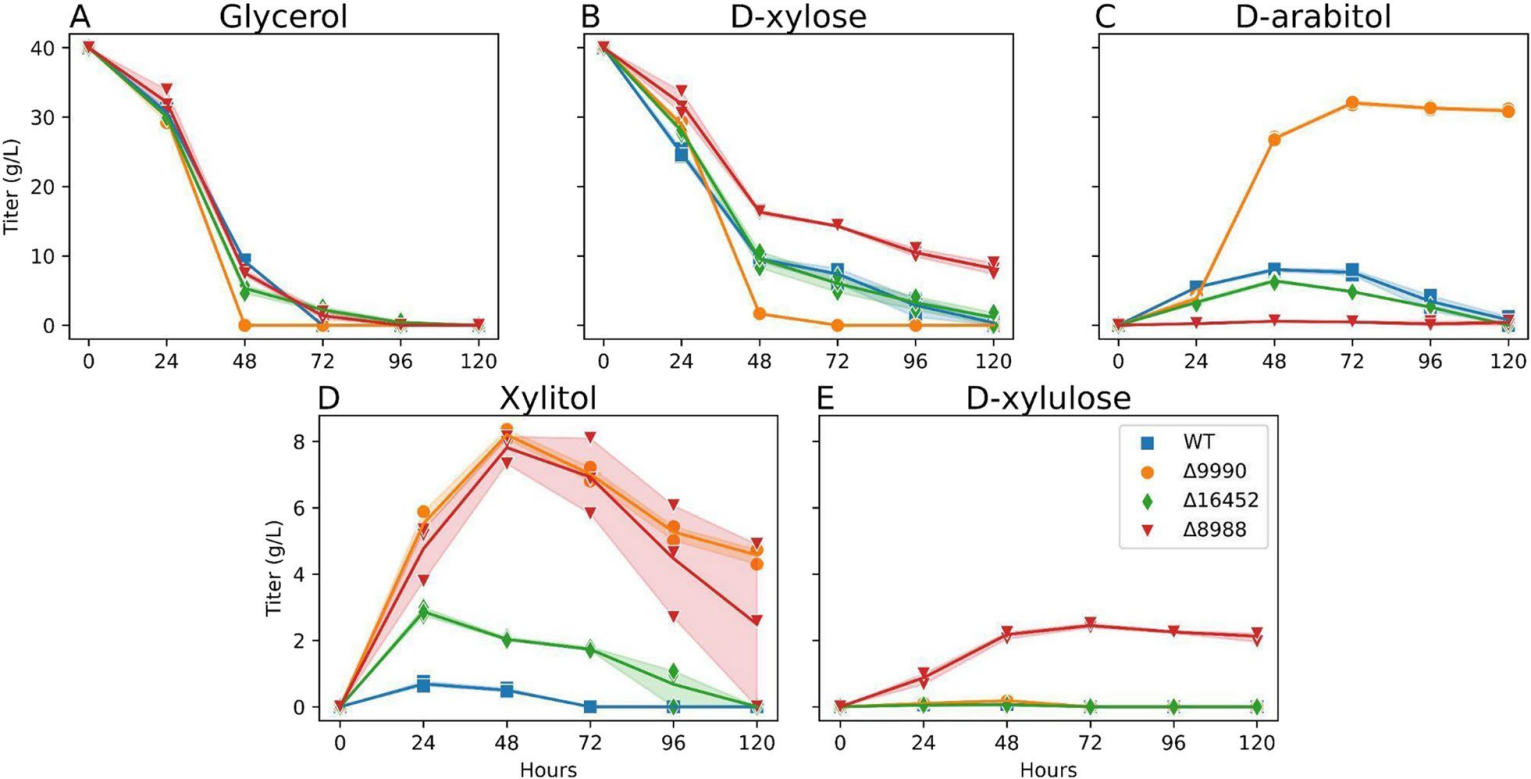
WT, ΔRTO4_9990, ΔRTO4_16452, ΔRTO4_8988 time-series measurements of supernatants grown on 40 g/L d-xylose + 40 g/L glycerol. WT did not produce any detectable d-arabitol or xylitol with glycerol as the sole carbon source. ΔRTO4_9990 is unable to grow on d-xylose (Fig. 6A); hence, all strains were supplemented with glycerol to aid biomass production. Solid lines are the average of 3 biological replicates; shaded regions indicate 100% percentile intervals

To complement growth phenotyping data, we collected pentose intermediate time-course data from select strains grown on 40 g/L d-xylose with 40 g/L glycerol (Fig. 9). ΔRTO4_8988 is the only strain that accumulates d-xylulose (Fig. 9E), supporting RTO4_8988 DA4DH activity (Fig. 3C). Glycerol consumption is uninhibited, but d-xylose utilization is heavily impacted; however, at peak titers (48 h), nearly 50% of consumed d-xylose is temporarily converted to xylitol, supporting XDH activity (Fig. 9D). In vitro characterization of *P. anomala* arabitol dehydrogenase (38% identify, 70% coverage to RTO4_8988) shows reversible DA4DH activity and irreversible XDH activity (xylitol to d-xylulose) [28]*.* After 48 h, though an additional 10 g/L d-xylose are consumed, 4 g/L of xylitol are also consumed. This may reflect increased expression of other enzymes in the pathway over time, shifting the rate-limiting step to d-xylose import or XR activity.

ΔRTO4_9990 converted the majority of d-xylose to d-arabitol with a small fraction to xylitol that was eventually consumed (Fig. 9C–D), consistent with RTO4_9990 encoding a DA2DH (Fig. 6). If RTO4_9990 additionally encoded for significant DA4DH activity, we would expect to see an accumulation of d-xylulose (similar to ΔRTO4_8988), but we do not (Fig. 9E). Curiously, ΔRTO4_9990 is able to consume both d-xylose and glycerol more rapidly than WT, hinting that RTO4_9990 itself might be one of the suspected rate-limiting steps in pentose metabolism. Lastly, ΔRTO4_16452 glycerol and d-xylose utilization were similar to WT (Fig. 9A–B); however, there was a modest, but significant temporal decrease and corresponding increase of d-arabitol and xylitol, respectively, relative to WT—supporting RTO4_16452 XDH activity (Fig. 9C–D). No excretion of d-xylulose was observed in ΔRTO4_16452, matching that of WT (Fig. 9E).

### Verification of arabitol enantiomer production

**Fig. 10:**
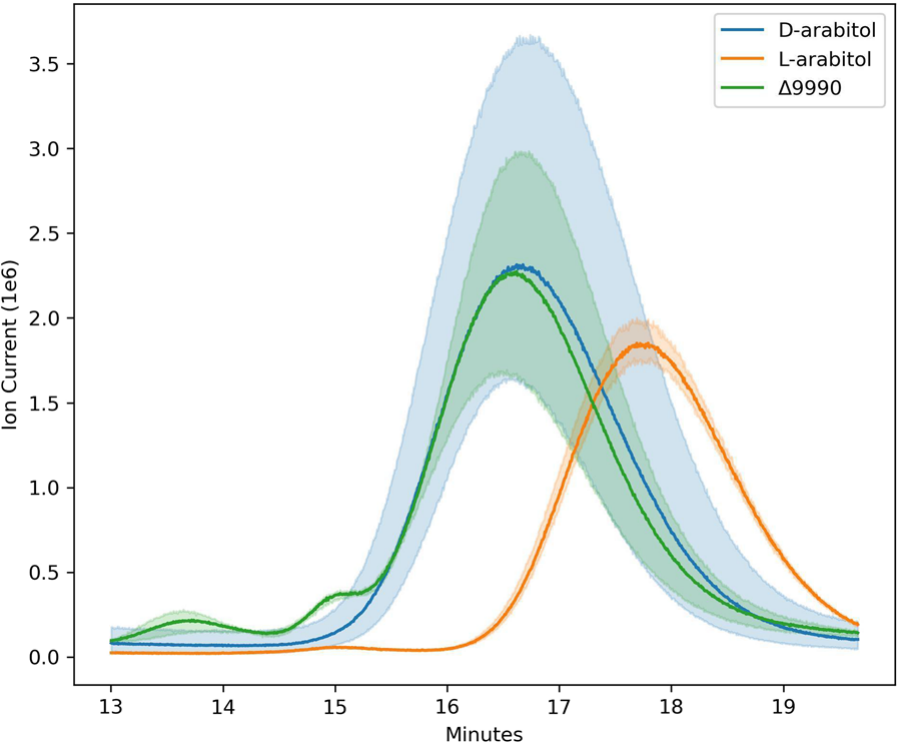
Derivatized d-arabitol and l-arabitol standards compared to derivatized sample supernatant of end-point culture of ΔRTO4_9990 grown on 40 g/L d-xylose + 40 g/L glycerol (Fig. 9). Solid lines are the average of 3 (biological) replicates; shaded regions indicate 100% percentile intervals

In fungi, few studies have definitively verified the actual production pathway of arabitol or distinguished the enantiomer produced [31], which is important for downstream applications [33]. Neither of these two inquiries have been satisfactorily investigated in *R. toruloides* or related *Rhodotorula* species; therefore, we performed chiral separations of d-arabitol, l-arabitol standards, and the supernatant from ΔRTO4_9990 grown on glycerol plus d-xylose (Fig. 9C) via gas chromatography-mass spectrometry (GC–MS; see methods). Indeed, Fig. 10 shows that the arabitol isomer produced from d-xylose is d-arabitol.

### Enzymatic d-arabitol-2-dehydrogenase redundancy

**Fig. 11:**
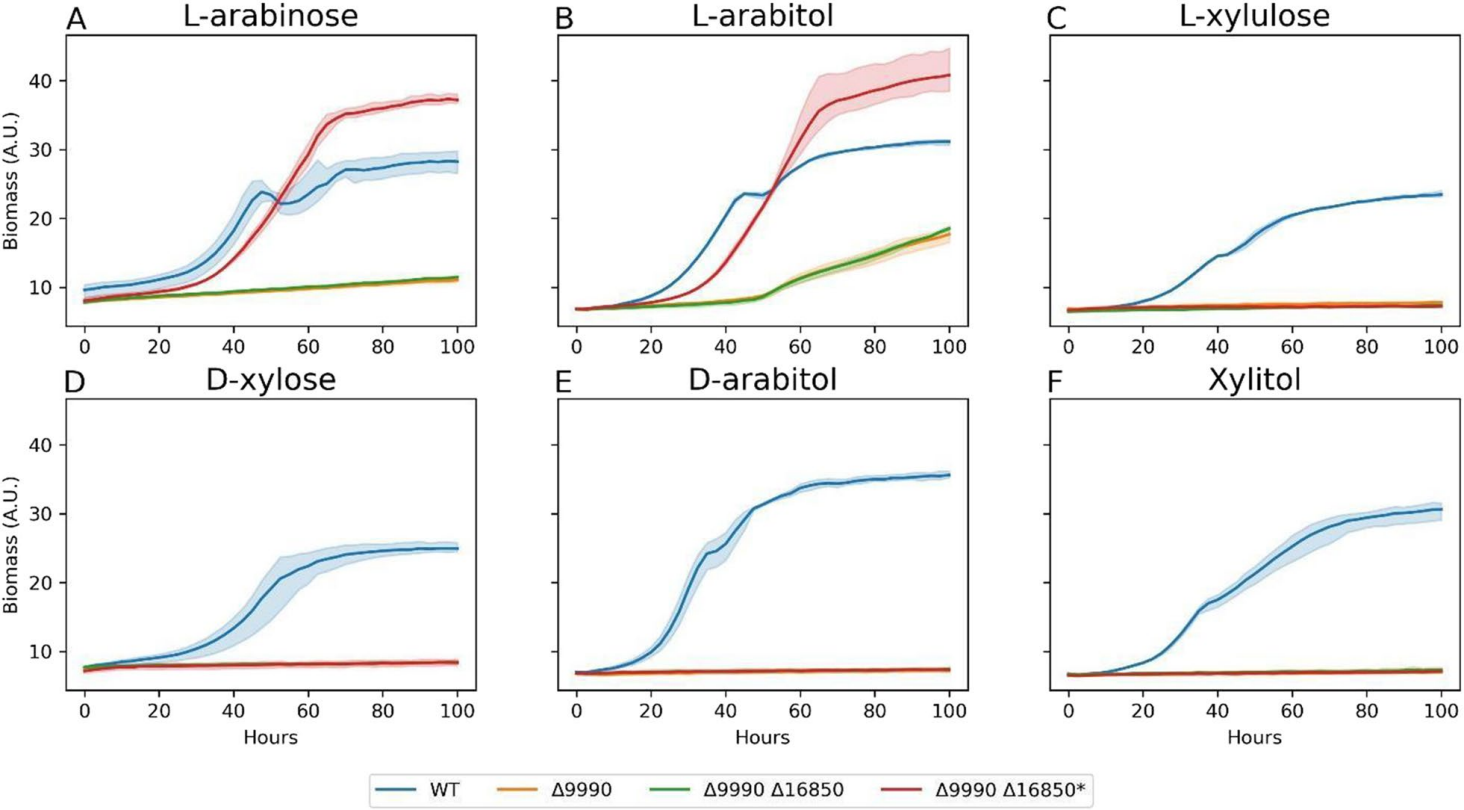
WT, ΔRTO4_9990, ΔRTO4_9990 ΔRTO4_16850, ΔRTO4_9990 ΔRTO4_16850* biomass growth curves on 10 g/L per sugar. Solid lines are the average of 3 biological replicates; shaded regions indicate 100% percentile intervals

**Fig. 12:**
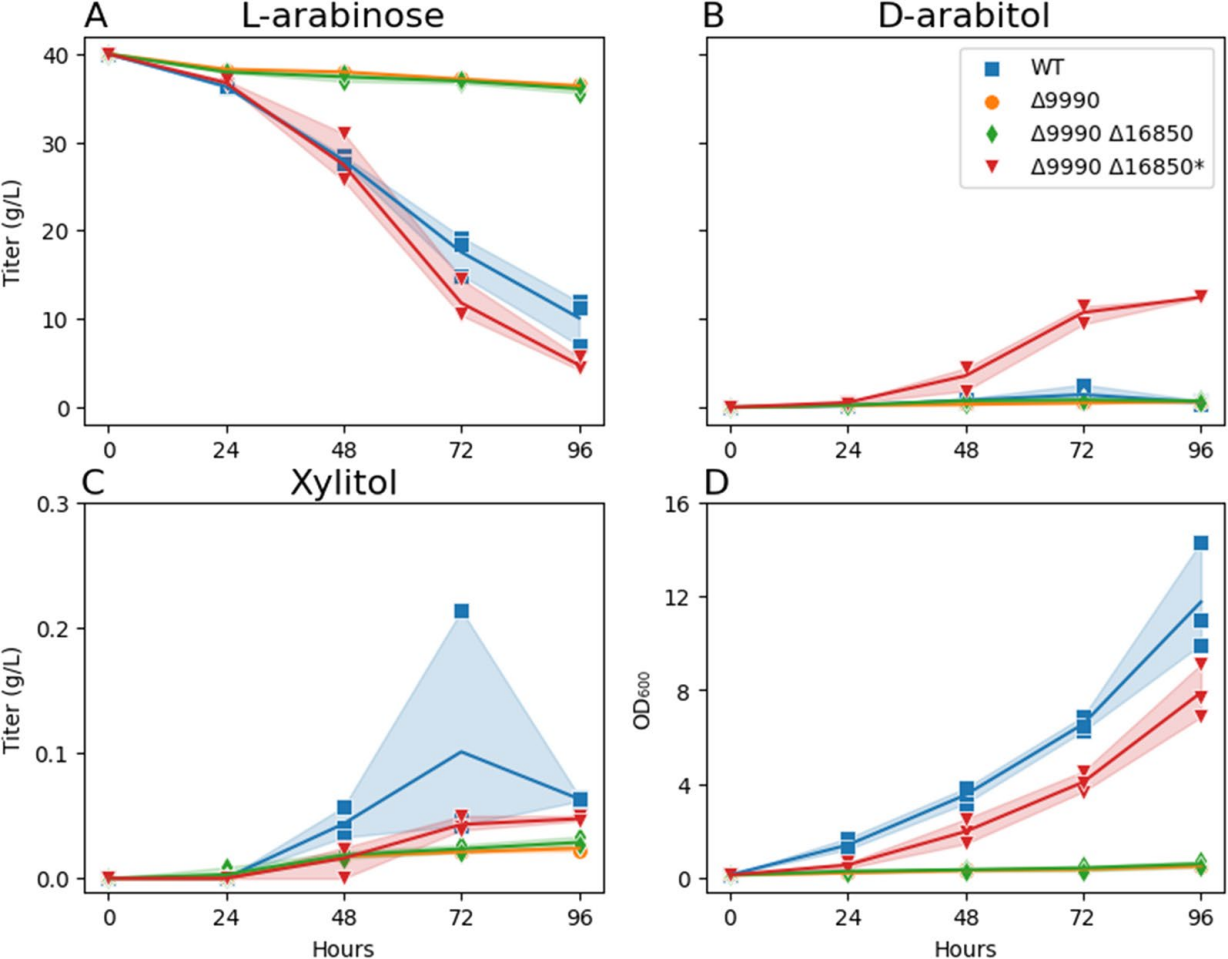
WT, ΔRTO4_9990, ΔRTO4_9990 ΔRTO4_16850, ΔRTO4_9990 ΔRTO4_16850* time-series supernatant measurements from cultures grown on 40 g/L l-arabinose. Solid lines are the average of 3 biological replicates; shaded regions indicate 100% percentile intervals

**Fig. 13:**
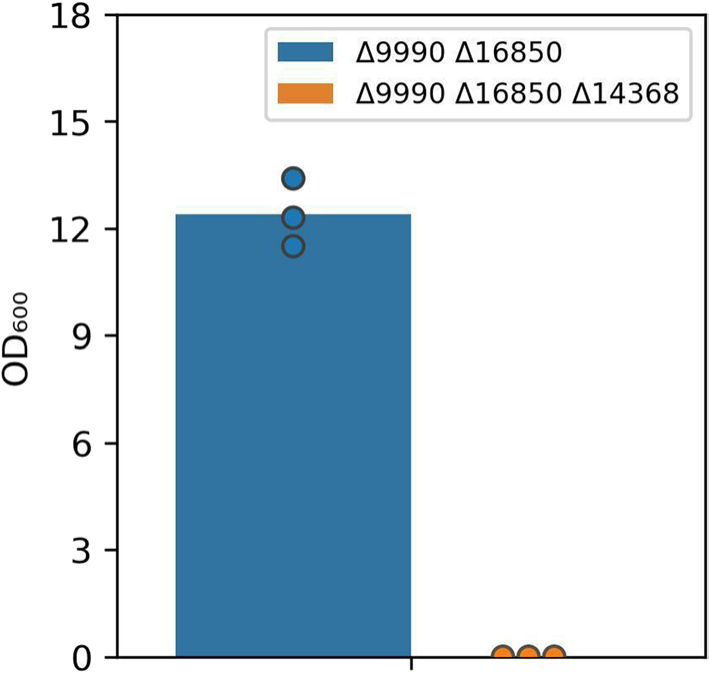
Averaged biological triplicate, end-point OD_600_ measurements of ΔRTO4_9990 ΔRTO4_16850 and ΔRTO4_9990 ΔRTO4_16850 ΔRTO4_14368 grown on 40 g/L l-arabitol

In Fig. 6, ΔRTO4_9990 did not grow on any substrate except for l-arabitol, with slow growth between 40 and 100 h (Fig. 6D), suggesting latent DA2DH redundancy in addition to activity encoded via RTO4_9990. In Fig. 9, a carbon balance between d-xylose, d-xylulose, d-arabitol, and xylitol was approximately closed until mid-run, then approximately 10% of carbon was unaccounted for by the end of the experiment. The onset of this carbon balance gap coincided with the onset of slow growth of ∆RTO4_9990 (Fig. 6D). To test if latent expression of RTO4_16850 was responsible for ∆RTO4_9990’s slow growth on l-arabitol, we constructed a double-deletion strain (i.e., ΔRTO4_9990 ΔRTO4_16850); however, mild growth was still observed on l-arabitol. ΔRTO4_9990 ΔRTO4_16850 was then adapted on 40 g/L l-arabitol (see methods) to generate ΔRTO4_9990 ΔRTO4_16850*. The evolved strain and controls were then cultured on 6 pentose substrates, including l-arabinose (Fig. 11). The evolved strain (ΔRTO4_9990 ΔRTO4_16850*) grew faster than the parent strain on both l-arabitol and l-arabinose, surpassing WT biomass yields with a similar growth rate. However, ΔRTO4_9990 ΔRTO4_16850* is still not able to grow on any other pentose substrate. Further, the evolved strain and controls were grown on 40 g/L l-arabinose, monitoring pentose intermediates (Fig. 12). Over 10 g/L of d-arabitol accumulated in cultures of the evolved strain. This, coupled with xylitol excretion, provides strong evidence of unknown redundant DA2DH activity alongside activity encoded via RTO4_9990. One potential homology-based candidate is RTO4_8988, the only gene that shows any similarity to RTO4_9990 (93% coverage and 40% identity); however, no growth defects were observed for RTO4_8988 mutants on d-arabitol in Fig. 3F. If the late growth on l-arabitol and l-arabinose is occurring through alternative DA2DH function, we would expect to see growth halted if RTO4_14368 is deleted in a ΔRTO4_9990 ΔRTO4_16850 background. Indeed, after 21 days of culturing on 40 g/L l-arabitol, no growth could be measured for the triple-deletion strain (ΔRTO4_9990 ΔRTO4_16850 ΔRTO4_14368), whereas significant growth was measured for ΔRTO4_9990 ΔRTO4_16850 (Fig. 13).

## Discussion

Recent studies of *R. toruloides* are concentrated on metabolic engineering, especially lipid production from biomass hydrolysates [67–69], with less attention to aspects of non-lipid metabolism [13, 15, 40, 49, 70, 71]. More of these studies are needed to identify rate-limiting steps, enzyme redundancy, and cofactor preference of major catabolic pathways to improve productivity, yields, titers, and efficiency of bioproduction. We present here a dataset probing metabolism of l-arabinose and d-xylose (abundant components of renewable biomass) in the non-model fungus *R. toruloides* which utilizes a non-traditional pathway for pentose metabolism, resulting in xylitol and d-arabitol accumulation from d-xylose.

**Fig. 14:**
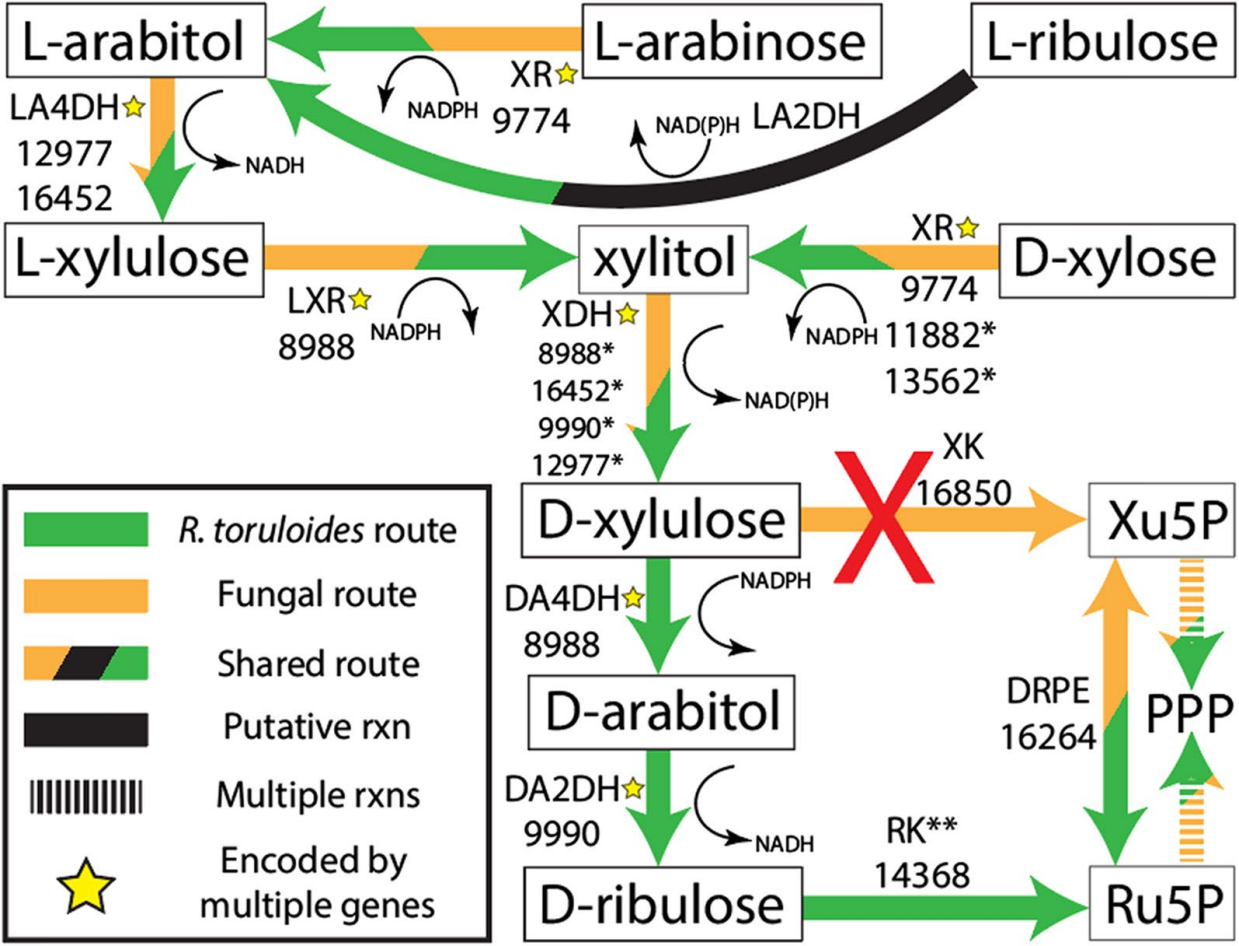
Gene-protein-reaction rules of putative l-arabinose and d-xylose metabolism in *R. toruloides* incorporating all data in this study. (*): Genes with annotation uncertainty due to insufficient evidence (see Table 1); (**): Evidence suggests RK activity is solely encoded by RTO4_14368; Some reactions have multiple enzymes (yellow stars) mediating catalysis, possibly encoded by genes outside the subset depicted here. *XR* broad-substrate-specificity d-xylose reductase, *LA4DH* l-arabitol-4-dehydrogenase, *LA2DH* l-arabitol-2-dehydrogenase, *LXR* l-xylulose reductase, *XDH* xylitol dehydrogenase, *DA4DH* d-arabitol-4-dehydrogenase, *DA2DH* d-arabitol-2-dehydrogenase, *RK* d-ribulose kinase, *XK* d-xylulose kinase, *DRPE* d-ribulose-5-phosphate 3-epimerase, *Xu5P* d-xylulose-5-phosphate, *PPP* pentose phosphate pathway, *Ru5P* d-ribulose-5-phosphate, *rxns* reactions, red ‘X’ denotes that *R. toruloides* does not carry flux via XK under conditions tested, despite possession of an XK-encoding gene, RTO4_16850

RNAseq, proteomics, and functional genomics [13], together with d-arabitol accumulation in two *R. toruloides* strains [14, 72], as well as documented d-arabitol production from non-l-arabinose sources in other yeasts [33], strongly suggest *R. toruloides* l-arabinose and d-xylose metabolism does not occur via canonical fungal XR, XDH, and XK. Our growth complementation data (Fig. 6) and d-arabitol accumulation data (Figs. 9 and 12) strongly support an alternative pathway through d-arabitol. Moreover, it is an absolute requirement for DA2DH activity encoded via RTO4_9990 to robustly metabolize any pentose substrates. Similarly, expressing a functional RK (encoded via RTO4_14368) is an absolute requirement to metabolize any pentose substrate studied (Figs. 7 and 8)—the only studied enzyme with no known functional redundancy. Lastly, the coordinated transcriptional upregulation of RTO4_14368 and RTO4_9990 strongly suggests simultaneous flux occurs through these steps during d-xylose and l-arabinose metabolism [13]. Figure 14 is an updated pathway reflecting all data collected in this study, accompanied by Table 1, a tabular summary of supporting data for all gene-protein-reaction rules.

**Table 1:** Summary of data supporting pentose gene-protein-reaction rules reflected in Fig. 14

Gene ID	Functions	Supporting Data (Grade)	Prev. Refs	Figure 14 inclusion	Remarks
N/A	DA2DH	Figure 11A, B (***) Fig. 12B (*) Fig. 13 (***)	N/A	Yes	Evidence of latent DA2DH activity; evolved strain produces d-arabitol
N/A	LA2DH	Figure 7 (***)	N/A	Yes	Gene(s) encoding this function have yet to be identified
9837	DA4DH	N/A	[13]	No	Single KO shows no growth defect
8905	DA4DH	N/A	[13]	No	Single KO shows no growth defect
14368	RK	Figures 7,8 (***)	[13]	Yes	Data suggests RTO4_14368 is the only RK-encoding gene
16850	XK	N/A	[13, 15]	Yes	Inactive under tested conditions in this study,but transcribed in presence of acetate [15]
13562	XR	N/A	[13]	Yes	Function unconfirmed in this study
11882	XR	N/A	[13, 40]	Yes	In [40], 11,882 has one-tenth the activity of 9774 on d-xylose
9990	DA2DH XDH	Figures 6F, 7, 9C, 10 (***) Fig. 9D (*)	[13]	Yes	Cannot rule out XDH function
8988	LXR XDH DA4DH	Figure 3D, E (***) Fig. 3B, 9D (*) Fig. 3C, 9E (***)	[13]	Yes	KO metabolite excretion patterns consistent with XDH activity
9774	XR	Figure 2 (*)	[13, 40]	Yes	Reduces l-arabinose, d-xylose, glyceraldehyde
16452	LA4DH XDH	Figure 5D (***) Fig. 9C, D (**)	[13]	Yes	KO metabolite excretion patterns consistent with XDH activity
12974	LA4DH XDH	N/A	[13]	No	Pseudogene; no growth defects with KO
12977	LA4DH XDH	Figure 5D (***) N/A	[13]	Yes	KO has slight growth defect on l-arabitol;cannot rule out XDH activity without metabolite profiling

Gene IDs are synonymous with their ‘RTO4_’ counterparts; ‘Functions’ column denotes the purported function(s) of the enzyme encoded by each gene; ‘Data (Grade)’ indicates which Fig(s) supports each function followed by a 3-tier asterisk system, a subjective rating of how well each piece of data supports each function (e.g., *** is strong support); Previous references that support functions are under ‘Prev. Refs’; ‘Fig. 14 Inclusion’ denotes which genes are included in the updated pentose model (i.e., Fig. 14). Corresponding supporting data for entries with multiple functions are separated by semicolons.  *XR* broad-substrate-specificity d-xylose reductase, *LA4DH* l-arabitol-4-dehydrogenase, *LA2DH* l-arabitol-2-dehydrogenase, *LXR* l-xylulose reductase, *XDH* xylitol dehydrogenase, *DA4DH* d-arabitol-4-dehydrogenase, *DA2DH* d-arabitol-2-dehydrogenase, *RK* d-ribulose kinase, *XK* d-xylulose kinase

Under d-xylose conditions, no evidence exists for expression of a functional *R. toruloides* XK (RTO4_16850) [13, 15]. This is corroborated by our inability to complement growth of ΔRTO4_9990 with RTO4_16850 expression under a strong endogenous promoter Tef1 [49]. Unexpectedly, XK is highly transcriptionally upregulated in the presence of acetate (whereas the rest of pentose metabolism is downregulated), but the corresponding protein abundance, function, and role XK plays regarding acetate metabolism is unknown [15]. Further investigation into RTO4_16850 functionality (e.g., in vitro kinetics or growth complementation in XK-deficient systems) is warranted. Nonfunctional or dormant pathways are not uncommon. In *Y. lipolytica*, WT is incapable of utilizing both d-xylose [73], but a study complementing mutant *E. coli* with genes encoding putative xylose-catabolizing proteins from *Y. lipolytica* demonstrated functional XR, XDH, and XK [74]. WT overexpression of endogenous XK and XDH resulted in robust growth on d-xylose, without the need for adaptation. Similarly, *Y. lipolytica* does not consume l-arabinose despite transcriptomics and enzyme activities showing a potential pathway may be active [75]. Culturing an engineered strain (that utilizes d-xylose) on a mixture of d-xylose and l-arabinose, a dramatic improvement in l-arabinose consumption was observed, suggesting l-arabinose catabolism can exist but is dormant due to inhibitory regulation, in addition to rate-limiting LA4DH activity.

Relative to Figs. 1, 14 shows a few alternative/putative pathways to metabolize d-xylose that have limited evidence in fungi, or are not consistent with our data. One example is xylitol conversion to xylitol-5-phosphate (X5P), reported in *Streptococcus mutans OMZ 176* [76], followed by X5P dehydrogenase (X5PDH) conversion to Xu5P prior to PPP entrance [77, 78]. Xu5P can be reduced via d-arabitol phosphate dehydrogenase (DAPDH) to d-arabitol-1-phosphate (DA1P) [78, 79], followed with dephosphorylation by broad-specificity polyol phosphate phosphatase (POPP) to d-arabitol, such as one from bacteria [80] or fungi [81]. The *S. cerevisiae* POPP identified from Xu et al. is active on d-glycerol-3-phosphate, ribitol-5-phosphate, and sorbitol-6-phosphate; however, with POPP active site structural similarity, the authors also speculate catalysis of d-arabitol-5-phosphate (DA5P), X5P, and erythritol-4-phosphate [81]. Analogously, fungi first reduce d-fructose-6-phosphate to mannitol-1-phosphate, then dephosphorylate to mannitol, as opposed to the reverse [82]. Finally, d-arabitol can arise from dephosphorylation of DA5P [20, 77–79].

l-arabinose and d-xylose metabolism are alternating series of reduction and oxidation steps, presenting a difficult task of cofactor balancing and redox homeostasis, a possible reason *R. toruloides*, *A*. *niger* [83], and other fungi have functional pentose enzyme redundancy [31]. Typically, different cofactor preference patterns exist for each step amongst molds and yeast [65], with exceptions such as XRs from 3 different yeasts (*S. cerevisiae*, *P. stipitis*, *Candida parapsilosis*) displaying unique cofactor preferences—solely NADPH, both, or mostly NADH, respectively [84–86]. Figure 14 shows the predominant cofactors participating in each step of the *R. toruloides* pathway as predicted by homology to characterized enzymes, but these predictions require experimental validation. Furthermore, in vitro characterization of enzyme kinetics, substrate preference, and cofactor usage might elucidate interesting selective growth of certain substrate-strain combinations tested such as ∆RTO4_8988 + OE XK, ∆RTO4_9990 + OE XK, or ΔRTO4_9990 ΔRTO4_16850* (Figs. 3B, 6B, 11C–F, respectively).

l-ribulose is readily metabolized by WT (Fig. 7) through a pathway yet to be elucidated. This may occur by isomerization to l-arabinose (via l-arabinose isomerase) or conversion to l-ribulose-5-phosphate (via l-ribulose kinase) (Fig. 1). Alternatively, l-ribulose could be converted to l-arabitol via an l-arabitol-2-dehydrogenase (LA2DH; Fig. 1), as demonstrated in purified extracts of the fungus *Penicillium chrysogenum* [87] and in the bacterium *Pantoea ananatis expressing* *xytf*[88, 89], with no clear orthologs in *R. toruloides*. Regardless of mechanism, ΔRTO4_9990 exhibits slow growth only on l-ribulose (not on d-ribulose), notably with the same final OD_600_ on l-arabitol (Fig. 7). In contrast, ΔRTO4_14368 neither grows on d-ribulose nor l-ribulose. Together, these data indicate that l-ribulose metabolism is upstream of RTO4_9990, likely part of l-arabinose metabolism (and possibly catalyzed via promiscuous LA2DH activity of unknown origin; Fig. 14). Secondly, this necessitates that d-ribulose is the product of DA2DH, with d-arabitol as the substrate (as there is no known reaction converting l-arabitol to d-ribulose).

## Conclusion

We have strongly improved upon and verified results from Kim et al. [13], positing that the primary route of *R. toruloides* d-xylose and l-arabinose metabolism proceeds through a common intermediate of fungal pentose catabolism—xylitol—followed by oxidation to d-xylulose, reduction to d-arabitol, oxidation to d-ribulose, phosphorylation to Ru5P, and entrance into the PPP. This route is most consistent with evidence of d-arabitol accumulation of ΔRTO4_9990 on d-xylose and the necessity of RTO4_14368 to metabolize any pentose intermediate. This unusual metabolism can be engineered to intricately control sugar and sugar alcohol product profiles of d-ribulose, d-arabitol, xylitol, and d-xylulose, four promising chemicals that can be made from sustainable biomass feedstocks [90]. A continued, multi-faceted approach to understand fundamental metabolism of *R. toruloides* and related fungi will help accelerate metabolic engineering efforts toward bioproducts by identifying potential rate-limiting steps and genes responsible for encoding enzymatic redundancy of major catabolic pathways.

## Methods

### Strains and sequences

*Rhodosporidium toruloides* (a.k.a. *Rhodotorula toruloides,* a.k.a *Rhodotorula graciis*) strain IFO 0880 (a.k.a NBRC 0880) was obtained from the Biological Resource Center, NITE (NBRC), Japan. All strains named in this work are available to order through the Agile BioFoundry parts registry at https://public-registry.agilebiofoundry.org. The registry website also hosts all applicable plasmid sequences. Applicable strains and plasmid sequences are listed by figure in Additional file 1. Protein identification numbers used in this manuscript are from the *R. toruloides* genome version 4, available on Mycocosm, the US Department of Energy Joint Genome Institute fungal genome repository [91]. Selection markers used in *R. toruloides* were hygromycin, G418, zeocin resistance cassettes using the *R. toruloides* Tub2 promoter and terminator (see sequences on the Agile BioFoundry parts registry).

For strains constructed by homologous recombination (e.g., full-deletion mutants), the parental strain was wild type. Homologous recombination and non-homologous end-joining (i.e., for generating randomly integrated mutants) was achieved by transforming *R. toruloides* via TDNA insertion with 1 kbp homology arms to the targeted locus by *Agrobacterium tumefaciens*-mediated transformation as described in [92]. Strain construction methods are listed for each strain in Additional file 1. For all deletion mutants, successful deletion was confirmed by diagnostic PCR at the altered locus. Plasmids with heterologous gene expression were codon optimized via the high-CAI method (i.e., the most used codons in *R. toruloides*). For construction of overexpression strains by random insertion, ~ 48 randomly selected transformants were screened for growth in liquid culture with 100 µg/mL of the appropriate selective agent, comparing growth to WT, and selecting the best-performing strain for further analysis.

### Media and growth conditions

All chemicals used in this study were from Sigma Aldrich unless otherwise stated. l-xylulose, d-xylulose, l-ribulose, and d-ribulose (XYU-009, XYU-001, RBU-005, and RBU-004, respectively) were purchased from Omicron Biochemicals. For regular strain maintenance and transformation, cells were grown in 10 g/L yeast extract, 20 g/L peptone, 20 g/L glucose (YPD). All strains were first grown on YPD agar plates (15 g/L agar), followed by picking of individual colonies to obtain biological replicates.

For Figs. 2, 3, 4, 5, 6, 7, 8, 9, 11, 12 and 13, cells were subcultured in YPD overnight, washed with water, inoculated at an optical density at 600 nm (OD_600_) of 0.1 (< 0.001 for Figs. 7 and 13) in 800 µL (5 mL Figs. 4, 12, 13) experimental medium and grown for the allotted time in the Figure (240 h or 21 days for Figs. 7 and 13, respectively) in a long-neck culture tube (Figs. 4, 12, 13) or a microtiter plate format: 48-well M2P Labs Flower Plate MTP-48-B (2000 µL total volume) at 1300 RPM agitation (1000 RPM for Figs. 7 and 9, 200 RPM for Figs. 4, 12, 13), 30 °C, and 85% relative humidity (50% for Figs. 4, 12, 13) in a BioLector Pro (BioLector 1 for Fig. 11) high-throughput microbioreactor (M2P Labs-Beckman Coulter) or in a 3 mm throw shaking incubator (Figs. 7 and 9). The M2P Labs Flower Plate allows for small-scale cultivation at high oxygen transfer rates [93]. For Figs. 6, 7, 11, 12 and 13, the final experimental media contained 1.7 g/L yeast nitrogen base without ammonium sulfate and amino acids (BD 233520), 5 g/L ammonium sulfate, 75 mM KH_2_PO_4_, 25 mM K_2_HPO_4_, 5 g/L each sugar (Figs. 7 and 11 10 g/L each sugar; 5 g/L d-xylose, 40 g/L l-arabinose, 40 g/L l-arabitol (Figs. 4, 12, 13, respectively)), pH 6.2. For Fig. 2, the experimental medium contained 1.7 g/L yeast nitrogen base without ammonium sulfate and amino acids (BD 233520), 40 g/L d-xylose, 203 mg/L MgCl_2_*6H_2_O, 246 mg/L MgSO_4_*6H_2_O, 2.5 mM KCl, 10 g/L yeast extract (Y1625), 3.5 mg/L ethylenediaminetetraacetic acid (EDTA), 27.8 mg/L FeSO_4_*7H_2_O, 70 mg/L C_6_H_8_O_7_*H_2_O, pH 5.6. For Figs. 3, 4, 5, 6 and 8, the experimental medium contained 1.7 g/L yeast nitrogen base without ammonium sulfate and amino acids (BD 233520), 5 g/L ammonium sulfate, 5 g/L each sugar (or 5 g/L d-xylose for Fig. 4), 100 µM, FeSO_4_, 400 µg/L thiamine HCl, 400 µg/L pyridoxine HCl, 180 mM KH_2_PO_4_, 20 mM K_2_HPO_4_, 176 mg/L nitrilotriacetic acid, 2.5 g/L MgSO_4_•7H_2_O, 2 g/L MgCl_2_•6H_2_O, 120 mg/L MnSO_4_•4H_2_O, 1 mM Na_2_SO_4_, 118 mg/L NaCl, 36 mg/L FeSO_4_•7H_2_O, 11.8 mg/L CoSO_4_•7H_2_O, 11.8 mg/L CaCl_2_•2H_2_O, 11.8 mg/L ZnSO_4_•7H_2_O, 1.2 mg/L CuSO_4_•5H_2_O, 1.2 mg/L AlK(SO_4_)_2_•12H_2_O, 11.8 mg/L H_3_BO_3_, Na_2_MoO_4_•2H_2_O, 2.5 mM KCl, pH 5.6. For Fig. 9, experimental medium contained 1.7 g/L yeast nitrogen base without ammonium sulfate and amino acids (BD 233520), 5 g/L ammonium sulfate, 40 g/L d-xylose, 40 g/L glycerol, 0.79 g/L complete supplement mix, 5 g/L yeast extract, 2 g/L MgSO_4_*7H_2_O, 100 mM KH_2_PO_4_, 10 M KOH-adjusted pH to 5. Periodic samples (40 uL, 250 µL, 250 µL for Figs. 4, 9 and 12, respectively) were aspirated for downstream analysis. Evaporation-corrected samples were taken and final OD_600_ measurements taken (Figs. 7 and 13).

### Adaptive evolution

Generation of the adapted strain ΔRTO4_9990 ΔRTO4_16850* began with a colony of ΔRTO4_9990 ΔRTO4_16850 isolated from plating on YPD agar. A single colony was picked and serially passaged every 3–5 days by transferring 5 µL of cells from the previous culture to a new well of 800 µL fresh media containing 1.7 g/L yeast nitrogen base without ammonium sulfate and amino acids (BD 233520), 5 g/L ammonium sulfate, 75 mM KH_2_PO_4_, 25 mM K_2_HPO_4_, 40 g/L l-arabitol, pH 6.2. Cells were grown in a microtiter plate format: 48-well M2P Labs Flower Plate (MTP-48-B) at 1000 RPM agitation, 30 °C, and 85% relative humidity in a 3 mm throw shaking incubator. Serial transfers occurred a total of 8 times. The final generation was stopped and plated onto YPD agar. Afterwards, to isolate a pure strain and test for stability of the phenotype, serial plating was completed on YPD agar by choosing a single colony each time, for a total of 5 times. 8 colonies were chosen from the final plating and tested in biological triplicate relative to WT on 40 g/L l-arabitol for improved growth. The most consistently reproducible isolate was chosen for further analysis and named ΔRTO4_9990 ΔRTO4_16850*.

### GC–MS analysis

End-point culture samples of ΔRTO4_9990 from Fig. 9 were processed and analyzed as described previously [31]. Briefly, 50 μl of each sample were evaporated to dryness under a stream of nitrogen, dissolved in dichloromethane (200 μl) and trifluoroacetic anhydride (400 μl), heated at 80 °C for 30 min, followed by nitrogen stream drying, and then redissolved in dichloromethane. GC–MS analysis was performed using a Chiraldex G-TA glass capillary column (ASTEC, Sigma 73035AST) and a single quadrupole Agilent GC–MS set at 70 eV. The helium carrier gas flow rate was set at 1 mL/min, and a four-step program was followed: 90 °C for 13 min, 0.8 °C/min up to 110 °C, 4 °C/min up to 180 °C, and 10 min at 180 °C. The injection port and ion source temperatures were maintained at 180 °C. Identification of the sample enantiomer was completed by retention time comparison to pure l-arabitol and d-arabitol standards.

### Sugar and sugar alcohol quantification

Sugars were quantified on a Dionex Ultimate 3000 system UHPLC (Agilent Technologies) using an Aminex HPX-87C column (Bio-Rad 1250095) and Thermo Scientific RefractoMax 520 Refractive Index Detector (RID) held at 35 °C. Prior to analysis, samples were diluted to 1:10 and filtered through a 0.45 µM polypropylene membrane microplate filter (Agilent 200983–100) by centrifugation at 3000 RCF for 3 min. Samples were run for 26 min using an isocratic HPLC-grade water mobile phase at 0.6 mL/min and 85 °C. Quantification was completed via peak area measurements compared to standard curves of pure compounds within their linear range of detection.

### Supplementary Information

**Additional file 1. **Strains used in this study with strain IDs and part numbers for relevant plasmids for retrieval on the Agile BioFoundry public registry (https://public-registry.agilebiofoundry.org).

## Data Availability

The datasets generated during and/or analyzed during the current study are available from the corresponding author on reasonable request. All strains named in this work are available to order through the Agile BioFoundry parts registry at https://public-registry.agilebiofoundry.org. The registry website also hosts all applicable plasmid sequences.
